# Seasonal Patterns of the Insect Community Structure in Urban Rain Pools of Temperate Argentina

**DOI:** 10.1673/031.009.1001

**Published:** 2009-03-30

**Authors:** M. Soledad Fontanarrosa, Marta B. Collantes, Axel O. Bachmann

**Affiliations:** ^1^ Departamento de Ecología, Genética y Evolución, Facultad de Ciencias Exactas y Naturales, Universidad de Buenos Aires, CONICET, Argentina; ^2^ Museo Argentine de Ciencias Naturales (MACN) CONICET, Argentina; ^3^ Departamento de Biodiversidad y Biología Experimental, Facultad de Ciencias Exactas y Naturales, Universidad de Buenos Aires, Argentina

**Keywords:** urban environments, insect assemblage, temporary pools, seasonality, hydroperiod

## Abstract

Temporary aquatic environments are widespread in the world, and although there are considerable regional differences in their type and method of formation they have many physical, chemical and biological properties in common. With the aim to increase knowledge of urban temporary pool fauna, the objectives of this work were to assess the seasonal patterns of species composition, richness, and diversity of the aquatic insect community inhabiting rain pools in urban temperate Argentina, and to identify the environmental variables associated to these patterns. Four temporary pools of an urban green space in Buenos Aires City were studied throughout a 1-year period. Eleven flood cycles with very varied hydroperiods and dry periods, mainly associated with rainfall, were identified. Insect species richness in these temporary urban pools, 86 taxa were documented, was found to be within the range reported for wild temporary water bodies of other regions of the world. The present results provide evidence for the existence of a clear link between habitat and community variability. Hydroperiod and seasonality were the main environmental factors involved in structuring the insect communities of the studied water bodies. Urban pools in green spaces have the potential to act to its dwellers like corridors through the urban matrix. Taking into account these characteristics and their accessibility, urban temporary pools can be considered as promising habitats for the study of ecological processes involving the insect community.

## Introduction

The comprehension of the processes that cause spatial and temporal dynamics of a community and the relationships between the characteristics of the habitat and species richness are important objectives in ecology (Spencer et al. 1999; Robertson 2000). However, to understand ecosystem functioning, we need to know not only how many, but also what kinds of species are present (Spencer et al. 1999). Both theoretical and empirical models suggested that habitat duration, or disturbance frequency, can mediate changes of relative importance in the biotic and abiotic processes that determine the distribution and abundance of the species within a wide spectrum of communities (Schneider and Frost 1996), including those of temporary pools (Wiggins et al. 1980; Wilbur 1997).

Temporary aquatic environments are widespread in the world, and although there are considerable regional differences in their type and method of formation they have many physical, chemical and biological properties in common (Williams 1996). These habitats impose rigorous conditions on the organisms living in them that must possess morphological, physiological and/or behavioural adaptations to survive (Hartland-Rowe 1966; Wiggins et al. 1980). The drying of the habitats could be considered a disturbance because it causes mortality that thereby decrease populations of aquatic organisms (Corti et al. 1997). Habitat desiccation also can influence biotic interactions by increasing the density of organisms, resulting in greater competition and other density-dependent effects (e.g., Aspbury and Juliano 1998), or by influencing colonization, survival, and developmental rates and strategies (Blaustein et al. 1999). Hydroperiod is important in the determination of community structure in temporary habitats (Wellborn et al. 1996; Spencer et al. 1999), and as the period between disturbances (drying) extends, more species can colonize these habitats and the interactions among the species can increase (Schneider and Frost 1996).

Blaustein and Schwartz (2001) broadly defined “temporary pools” to include any habitat that intermittently has standing water and that, once inundated, holds water long enough for some species to complete the aquatic phases of their life cycle. This definition includes water bodies that might be classified elsewhere as temporary lakes, temporary ponds, or phytotelmata. In addition, these authors state four compelling reasons to study the ecology of temporary pools: (1) habitat ephemerality is a common problem for organisms in a variety of habitats aside from temporary pools; (2) temporary pools provide highly tractable systems for addressing basic ecological questions; (3) temporary pools are breeding habitats for many medically important species; and (4) the unique communities found in temporary pool habitats are in need of conservation and protection. Studying temporary pools has the advantagesthat the main disturbance (drought) can be easily identified and that habitat duration can be quantified in a simple way. The community of a pool shares the same potential assemblage of organisms of neighbouring habitats, which are influenced by similar climatic and edaphic conditions in the region (Schneider and Frost 1996).

Freshwater habitats, particularly temporary and permanent pools, natural or artificial, are characteristic in urban green space. Urban areas often have high diversity of habitat types and species partly as a result of diverse human activities, but urbanization and human activities are also a threat to several natural habitats and species (Niemelä 1999). The consequences of urbanisation include changes in the richness, composition, and individual species abundance of animal and plant assemblages (Smith et al. 2006). Undeveloped land in towns and cities that supports vegetation and has freely draining surfaces, generically termed ‘green space’, can reduce the impact of urbanisation on temporary pools. Green space has the potential to lessen detrimental effects on species assemblages by preserving or creating habitat, and by retaining corridors through the urban matrix (Smith et al. 2006). The loss of urban green space jeopardizes the overall biodiversity of urban and sub-urban areas, and forces us to consider the importance of preserving urban nature more carefully in the planning process (Yli-Pelkonen and Niemelä 2005).

With the aim to increase knowledge of urban temporary pool fauna, the objectives of this work were to assess the seasonal patterns of species composition, richness, and diversity of the aquatic insect community inhabiting rain pools of urban temperate Argentina, and to identify the environmental variables associated to these patterns.

## Materials and Methods

### Study area

The city of Buenos Aires is located at 34°35′ S, 58°29′ W, at 25 m.a.s.l., on the right margin of the río de la Plata, an estuary of approximately 50 km of width in this section. The diameter of this almost circular city is approximately 16 km and its surface area covers ca. 204 km2. Its population of 2.8 million inhabitants constitutes the core of a huge megalopolis of 12.5 million people (INDEC 2001). The climate is humid temperate, with four definite seasons, mean annual precipitation of 1076 mm and mean annual temperature of 17.4°C (Instituto Geográfico Militar 1998).

The sampling sites are located in one of the largest (≈ 80 ha) green spaces of the city, known as “Parques de Palermo”. This parkland is used as a recreational area by many sportsmen, dog trainers and the public. The area is cleaned by the customary collection of garbage and lawn cutting. Temporary pools in the parkland are exclusively formed by rainwater accumulation in soil depressions. Four temporary pools (I, II, III, and IV) that were very close to each other, were selected for the present study. Except for flooded grass, these environments lack any kind of aquatic vegetation, indicating their ephemeral nature. The studied pools are mostly exposed to the sun, except for a few hours a day, when they are in the shade of nearby trees.

### Sampling protocol

The samples were taken from October 2001 through October 2002, with a sampling frequency of three days per week, starting from the formation of the water body until it was completely dry. On each sampling date, the pools were characterised by the following variables: a) percentage of herbaceous vegetation cover, b) water and air temperature, c) maximum and mean depth (averaging at least five different points), and d) surface area. The latter variable was estimated by measuring the maximum length and width of the pool, and then calculating the flooded percentage of this rectangular area. Daily mean, maximum and minimum temperatures, and daily accumulated rainfalls, were registered at a meteorological station located less than 1 km from the study area (data provided by the Servicio Meteorológico Nacional). The samples were collected with hand nets (350-µm mesh size) of two sizes of mouth opening (7.5 × 6 cm and 10 × 7.5 cm) selected according to mean depth and pool size. The sampling effort in each pool, considered as the total number of 1 -m long net sweeps, was proportional to surface area, and adequate to detect more than 70% of species present, based on preliminary yield/effort curves. With the aim of including the diversity of the different microhabitats of the pool, samples were taken both in the margin and centre, and in areas with and without vegetation. The samples were immediately fixed *in situ*, in 80% ethanol to avoid predation.

All the specimens collected were identified to the lowest taxonomic level as possible, also identifying their developmental stages (larva, pupa and adult) and larval stages when possible. Taxonomic identifications were performed using the appropriate systematic keys and specialised literature on the local fauna: general insects, (Merritt and Cummins 1984; Lopretto and Tell 1995); Coleoptera, (Archangelsky 1997; Grosso 1993; Oliva et al. 2002); Diptera, (Alexander and Byers 1981; Downes and Wirth 1981; James 1981; Darsie 1985; Knutson 1987; Paggi 2001; Rossi et al. 2002); Ephemeroptera, (Domínguez et al. 1994; Domínguez et al. 2001); Heteroptera, (Schnack 1976; Bachmann 1981; Bachmann and López Ruf 1994; Estévez and Polhemus 2001); Odonata, (Rodrigues Capítulo 1992); Trichoptera, (Angrisano and Korob 2001).

### Data analysis

Hydroperiod (Hyd) is defined as the time elapsed since the pool formation until the corresponding sampling day, while total hydroperiod (THyd) is the time elapsed from the formation of the pool until it dries completely, in both cases expressed in days. On the other hand, flood cycle is defined as the period elapsed from the formation of the water bodies (or starting day of the rainy period) until the day when all the pools dry up. The dry period before pool formation is also expressed in days. Origin rains are defined as the amount of pluvial precipitation occurring in one or more consecutive days, which originate pools. Complementary rains are the rainfalls that occur subsequent to pool formation and before it dries out, thus prolonging the hydroperiod of the water body. For each pool, Hyd and THyd, the community variables calculated were species richness (as the number of taxa), the Shannon Diversity Index, and evenness which is based on the Shannon Index (Zar 1999). Relative abundance was expressed as numbers of individuals per litre.

The non parametric Spearman rank correlation was used to evaluate possible relationships between different environmental variables, and between richness (or diversity) and THyd and the maximum flooding area (Zar 1999).

To determine the relative importance of the environmental variables on the species distribution, Canonical Correspondence Analysis (CCA, using CANOCO program, version 4, by ter Braak and Smilauer 1998) was performed. Forward analysis of variables was used in all cases, considering the explained percentage of the variance and its significance. The Monte Carlo test was used for the statistical validation of the association between the ordination values of species, samples and environmental variables. A total of 199 iterations were performed for this test and considered to be significant at p ≤ 0.05. To diminish the weight of extreme values of relative abundance, they were log-transformed as log_10_ (×+1) (ter Braak and Verdonschot 1995). The environmental variables considered for the analysis are summarised in Table 1.

## Results

### Habitat variability

**Figure 1.**
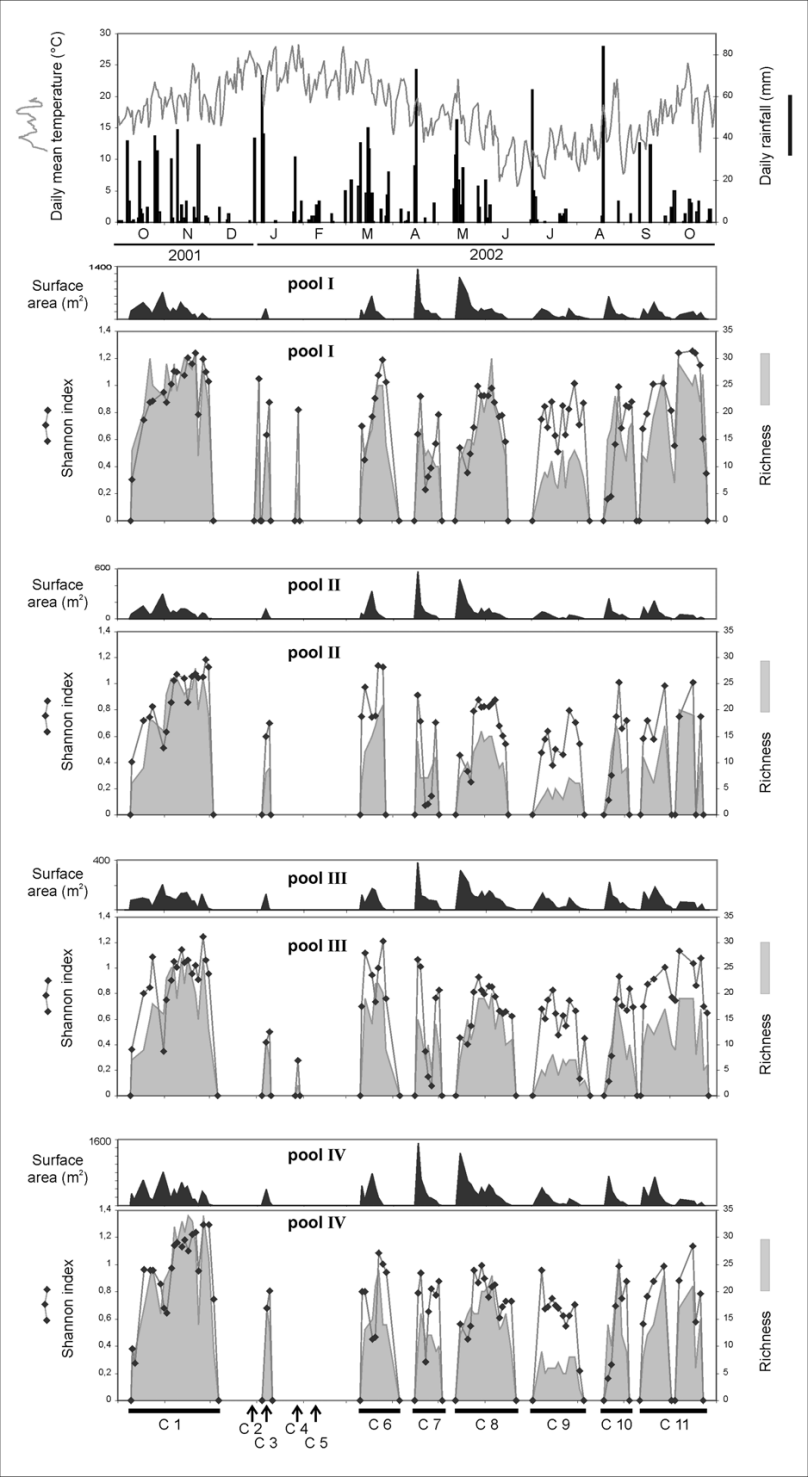
Daily mean temperature and daily rainfall recorded during the study period in Buenos Aires City. Surface area, Shannon Diversity Index and species richness registered during each flood cycle in each one of the studied pools. C= flood cycles (I to II).

The physical conditions of the pools were not stable over time. A total of 11 flood cycles, with THyd ranging from 2 to 59 days (mean = 28), were identified. The THyd of the summer cycles were the shortest ones (mean = 5, minimum = 2, maximum = 8 days), while the spring cycles were the longest ones (mean = 51, min = 43, max = 59 days). Dry periods ranged from a few days to almost two months. The maximum surface area of two of the pools was much larger than in the other two (pool IV: 1520 m^2^, I: 1330 m^2^, II: 570 m^2^, and III: 380 m^2^) (Figure 1). The maximum depth registered attained 34 cm (mean ±SE = 10.55 ±5.47, median = 9, Quartil_1_ = 7, Q_2_. = 13.3). Water temperatures fluctuated between 3.2°C and 30.6°C. THyd was positively and significantly correlated (r_s_ = 0.85, p < 0.001) to complementary rains but not to origin rains (r_s_ = -0.1, p = 0.57). The same result was observed for the pools individually considered. Also, THyd was negatively and significantly correlated to mean water temperature (r_s_ = -0.35, p < 0.04), and to median and minimum air temperature (r_s_ = -0.47 y r_s_ = -0.50, p < 0.001). However, considering each pool individually, THyd was never correlated to water and air temperature (mean, minimum or maximum).

**Table 1.**
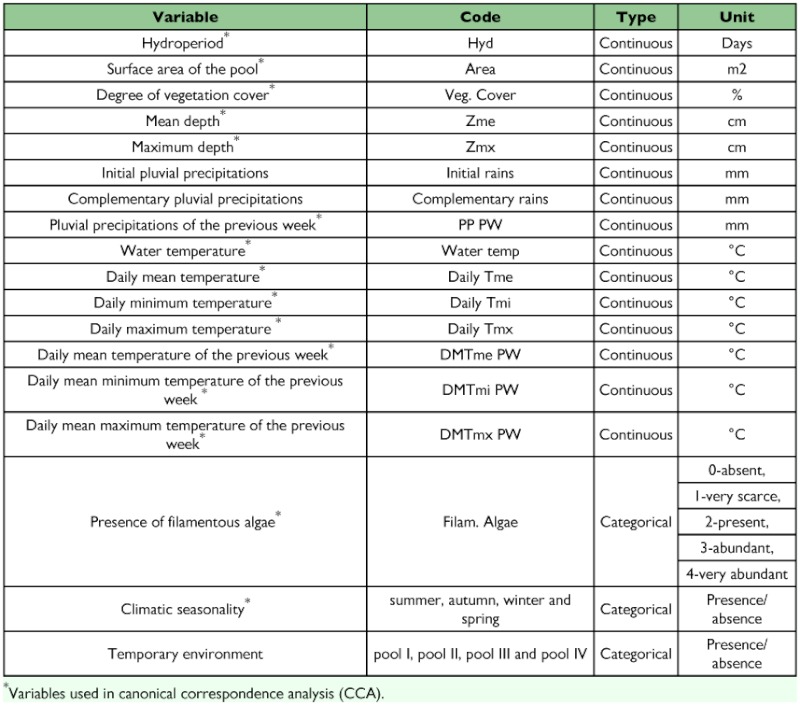
Code, type and unit for each variable included in the analyses.

On each sampling date, Hyd was negatively and significantly correlated to surface area (rs = -0.34, p < 0.001) and to maximum and mean depth (r_s_ = -0.39 and r_s_ = -0.44, respectively, p < 0.001). Surface area was positively and significantly correlated to maximum and mean depth (r_s_ = 0.88 and r_s_ = 0.82, respectively, p < 0.001), and to PP PW (r_s_ = 0.53, p < 0.001). Air and water temperatures were correlated neither to Hyd nor to surface area. The same results were obtained for each pool separately.

### Community structure

The overall species richness was 86 and the overall Shannon index diversity was 1.20. A total of 75374 individuals belonging to six insect orders were identified as follows: 32 morphospecies in 7 families of coleopterans, 28 in 10 families of dipterans, 20 in 10 families of heteropterans, 4 in 4 families of odonates, 1 genus of ephemeropterans, and 1 genus of trichopterans (Appendix). The total relative abundance of captured individuals of each order was: Diptera 42%, Coleoptera 23%, Heteroptera 19%, Ephemeroptera 12%, Odonata 3%, and Trichoptera 0.006%. Other faunal elements that were recorded but not included in the present analyses were cladocerans (*Daphnia* spp., *Moina* spp., Chydoridae, Macrothricidae, Sididae), cyclopoid and calanoid copepods, ostracods, conchostracans, mites, collembolans, gastropods, oligochaetes, tadpoles, and tintinnids (occasionally).

**Figure 2.**
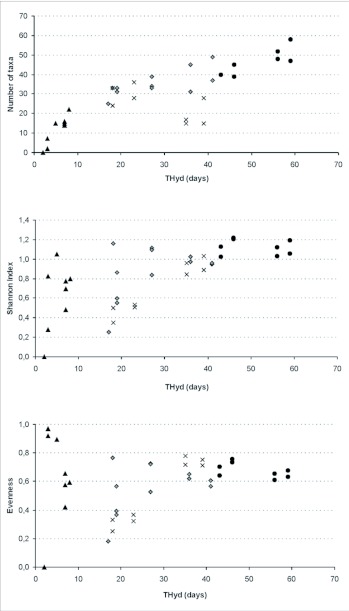
Number of taxa (richness), Shannon Index and evenness of the aquatic insect community estimated for each THyd (total period of inundation of each pool) during summer (triangles), autumn (rhombus), winter (x) and spring (circle).

Richness, species diversity and evenness varied throughout the study period (Figure 2). The highest richness and diversity corresponded to spring cycles and to some autumn cycles. The lowest richness and highest evenness were registered during the summer. THyd was positively and significantly correlated to richness (r_s_ = 0.81, p < 0.001) and diversity (r_s_ = 0.63, p < 0.001). On the other hand, the greater surface area reached on each flooding event was correlated to richness (r_s_ = 0.36, p < 0.03) but not to diversity (r_s_ = 0.20, p = 0.25).

The flooded surface area, richness and diversity registered in each pool differed from one flood cycle to the other (Figure 1). Each cycle was characterised by a rapid initial increase of richness and diversity, followed by fluctuations that end in a marked drop of these values on the final dates.

**Figure 3.**
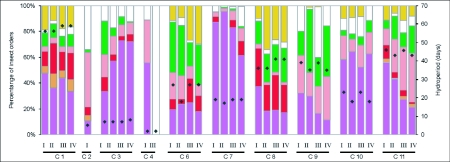
Percentage of insect orders (immatures and adults discriminated) and hydroperiod registered during each flood cycle in each one of the studied pools. Purple = immature Diptera, orange = immature Odonata, red = immature Ephemeroptera, green = immature Coleoptera, pink = adult Coleoptera, yellow = immature Heteroptera, and white = adult Heteroptera. I, II, III and IV indicate pool number; C= flood cycles (I to II).

Regarding the species composition of the different insect orders, dipteran larvae represented the most important fraction of the community in those pools having the shortest THyd. The extremely short summer cycles (2 and 4) were an exception, since adult coleopterans and heteropterans were the characteristic groups. During prolonged flooding episodes a higher number of taxonomic groups were more evenly distributed (Figure 3).

### Taxa seasonality and canonical correspondence analysis

Monthly relative abundances of the collected taxa are summarised in the appendix. Among dipterans, the number of Chironomidae and Culicidae larvae widely exceeded the other dipteran families except during the summer months. Among chironomids, Chironominae were dominant at the beginning of the autumn, Tanypodinae dominated towards the end of this season, and Orthocladiinae prevailed in the winter, while all three subfamilies were equally abundant in the spring. Muscidae and Ephydridae were the best-represented families during the summer. The presence of Sciomyzidae and Stratiomyidae was clearly seasonal, presenting maximum abundances in October and November. The highest biodiversity at the family level was recorded in the spring.

Among coleopterans, the best represented families were Dytiscidae and Hydrophilidae. Within the former, the larvae of *Rhantus* sp. and Bidessini (assigned to *Liodessus* sp.) were registered throughout the whole study period and were the main representatives of this group in the winter. The adults of *Rhantus signatus* were present on a few occasions while the adults of *Liodessus* sp. were detected all year around. The second family (Hydrophilidae) was also found throughout the whole study period, mainly represented by larvae and adults of *Tropisternus, Berosus* and *Enochrus*. Among coleopteran families, Noteridae were third in importance. The larvae of this family were only present in the spring and the autumn.

Among heteropterans, the adults of *Sigara platensis* clearly exceeded the number of adults of all other species of this order, and the same occurred with their larvae (Appendix). Adult heteropterans were regularly present throughout the year, while the larval stages were mainly found in the spring and the autumn with the exception of the larvae of *Sigara* spp. and *Notonecta* spp., which were present during most of the study period.

The largest relative abundance of Ephemeroptera larvae occurred in the spring and the autumn, while Odonata larvae were only abundant during the spring cycles.

**Figure 4.**
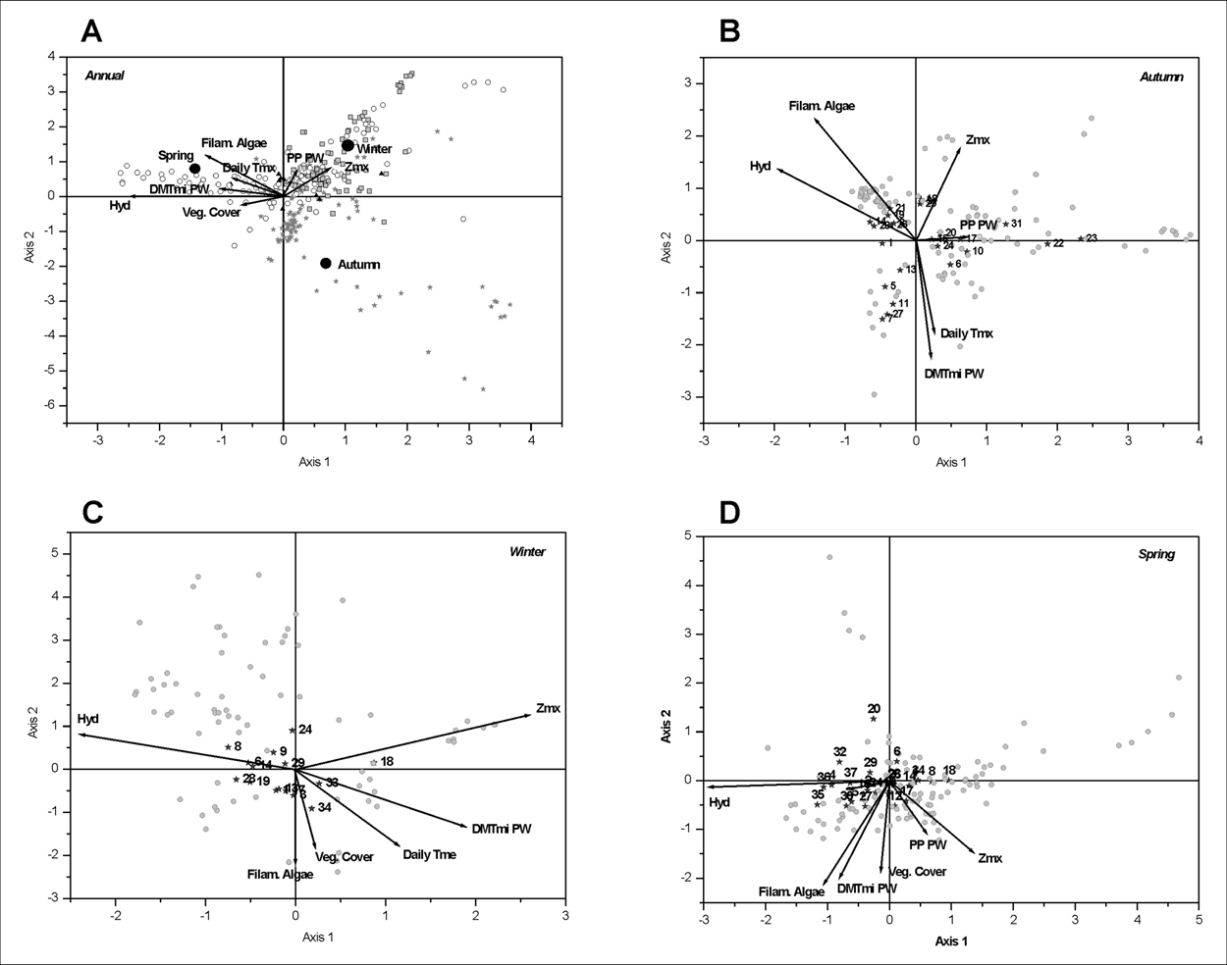
Canonical correspondence analysis (CCA) ordination graphic. Arrows = quantitative environmental variables. Solid circles = nominal environmental variables. Only the variables significantly associated with axes 1 and 2 were plotted. A: Annual. Samples corresponding to the autumn (stars), winter (squares), spring (open circles) and summer (triangles). B: autumn, C: winter, D: spring (samples = circles, species = stars, numbers indicate the taxa enumerated in the appendix).

Considering the relative abundance of the different species, the CCA showed a clear correspondence with the environmental variables selected for the annual overall data and for each season (Table 2). The Monte Carlo test was significant (p < 0.005) for the first canonical axis and for the whole set of axes in all analysed cases. Table 2 shows the entry order of the environmental variables that were significantly incorporated to the models formulated. For the annual overall data, Hyd and seasonality were the most important environmental variables in explaining data distribution (Figure 4). No environmental variable was significantly incorporated to the statistical model by the CCA for the summer data. For the three climatic seasons analysed, the statistical models formulated shared the following four variables: Hyd (hydration), DMTmi PW (a composed variable that synthesises the climatic conditions of the previous week), and Zmx (maximum depth).

**Table 2.**
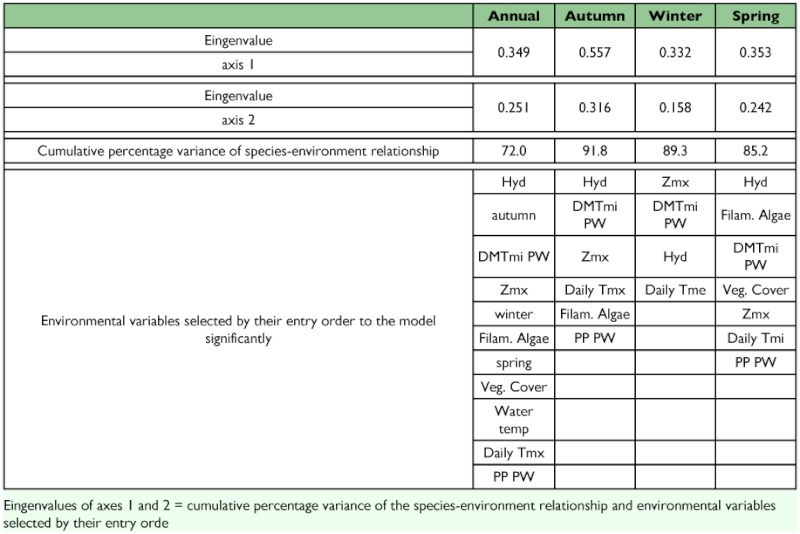
Results of canonical correspondence analysis (CCA) for the annual and seasonal analysis.

The Appendix enumerates the most important taxa for the performed CCA. The relevant taxa exclusively present in the autumn were the larvae of *Lancetes* sp. and *Vatellus* sp., the adults of *Berosus* sp., *T. setiger* and *B.fuscipennis*. The adults of *Desmopachria concolor* and *N. sellata* were the most important taxa during the winter. The spring was mainly characterised by the presence of larvae of different orders, that were found exclusively during this season: Sciomyzidae (Diptera); *Desmopachria* sp., *Laccophilus* sp. and *Paracymus* sp. (Coleoptera); *Notonecta* sp., *Hydrometra* sp. and *Microvelia* sp. (Heteroptera); and the larvae of both Odonata suborders.

## Discussion

In general, these results suggest that aquatic insects inhabiting urban temporary pools in a temperate region form part of a complex ecosystem in which the habitat and the insect community vary in both space and time. Some factors involved in the dynamics of the ecosystem respond to local features (e.g. pool depth), while others depend on the regional characteristics (e.g. temperature and rainfall).

### Habitat variability

The urban pools included in the present study are exclusively formed by rainfall and are characterised by drying out regularly. The flood cycles registered were shorter than those reported by the literature on temporary aquatic environments (e.g. Wiggins et al. 1980; Collinson et al. 1995; Williams 1996). In the temperate regions of the Northern Hemisphere, Wiggins et al. (1980) distinguish two types of predictable pools: vernal (spring filling) and autumnal pools. In temperate regions of the Southern Hemisphere that have winter rains and moderate temperatures, winter rather than vernal pools tend to occur (Lake et al. 1989). Conversely, in warm-temperate coastal regions of New South Wales, having both summer and winter rainfalls, pools may have an irregular filling pattern (Bishop 1967a, b). In coincidence with the observations by the latter author, the formation pattern of the pools of Buenos Aires, a city located in a humid-temperate region, is unpredictable in time (i.e. depending on the meteorological conditions) but predictable in space (i.e. they occur in the same depressions of the soil). The strong correlation between total hydroperiod and complementary rains indicates the importance of this water source for the permanence of these environments.

The observed relationships between hydroperiod, surface area and pool depth can be explained in terms of desiccation, i.e. as days pass, both surface area and depth diminish. Other studies carried out in different regions also report a strong relationship between water surface fluctuation and pluvial precipitation (Ebert and Balko 1987; Lake et al. 1989; Ward and Blaustein 1994; Bazzanti et al. 1996). Morphometric parameters such as depth, surface area, basin shape, and volume clearly affect the water balance and hydroperiod of ephemeral pools (Brooks and Hayashi 2002).

Temperature is a very important variable affecting temporary aquatic habitats, not just on a seasonal basis, but also on daily or even hourly periods (Williams 2006). Considering the total hydroperiod for the complete data set (n = 36) we observed a negative correlation between this variable and water and air temperatures, thus indicating that at higher temperatures total hydroperiods are shorter.

### Community structure

The 86 insect taxa registered in the studied urban pools corresponds to the values reported for wild temporary environments of different regions (Kenk 1949; Bazzanti et al. 1996; Williams 1996; Williams 1997; Lake et al. 1989; Boix et al. 2001; Graham 2002). Published observations on temporary environments worldwide report that aquatic insects constituted a very important portion of the animal community of these habitats (Kenk 1949; Lake et al. 1989; Bazzanti et al. 1996; Boix et al. 2001). Although in the present study the relative abundances of other taxonomic groups were not estimated, preliminary observations suggest a similar trend in our study area (personal observations). Similarly, the community composition matches previous studies that also reported Coleoptera, Diptera and Heteroptera as the best-represented insect orders (Kenk 1949; Bazzanti et al. 1996; Williams 1997; Fischer et al. 2000; Boix et al. 2001). Considering these three orders, maximum richness tends to concentrate in a few families, namely: dytiscids and hydrophilids among coleopterans, chironomids and culicids among dipterans, and corixids and notonectids among heteropterans (Boix and Sala 2002). Although chironomid larvae were identified to the subfamily level, our observations suggest the existence of several morphotypes within each subfamily; therefore, the richness contributed by this group would be higher than the estimated values. Among heteropterans, an important number of belostomatid species was also observed; however, only *B. elegans* was frequently collected in high numbers. Among the orders represented by a few taxa, trichopterans may be considered uncommon in these temporary environments, while ephemeropterans were frequently found (Boix and Sala 2002). Similarly, *Oxyethira* sp. was registered in low frequency and abundance, while *Callibaetis* sp., a genus previously cited from temporary habitats (Wiggins et al. 1980; Graham 2002), was highly frequent and abundant.

As already reported by previous studies, we found a positive correlation between flooded area and species richness (Roth and Jackson 1987; Ward and Blaustein 1994; March and Bass 1995; Bazzanti et al. 1996; Spencer et al. 1999). Other authors found that species richness is more sensitive to factors associated with water duration rather than with pool size (Ebert and Balko 1987). This increase in the species richness during longer hydroperiods was also observed by other authors in different temporary environments (Stout 1964; Nilsson and Svensson 1994; March and Bass 1995; Bazzanti et al. 1996; Schneider and Frost 1996; Spencer et al. 1999).

Conversely, diversity was never correlated to the surface area of the pools but to hydroperiod, thus suggesting that the complexity of the community structure increases with time. At longer hydroperiods, more species will be able to complete their development and maintain viable populations. In particular, for those species that disperse by flight as adults, longer pool permanence also implies a longer time available for colonization (Spencer et al. 1999). Hydroperiod is the main factor in determining the faunistic composition and community structure of temporary aquatic environments (McLachlan 1985; Jeffries 1994; Schneider and Frost 1996; Wellborn et al. 1996; Boix et al. 2001).

In most of the flood episodes studied, richness and diversity showed a rapid increase during the first days, followed by smooth oscillations. A dramatic reduction of these values occurred during the last days before the desiccation of the pools. Our results do not provide evidence that species richness or community composition may attain a stable point or equilibrium point. Temporary pond succession does not reach a stable climax and is definitely non-equilibrial, in this sense it resembles succession patterns like those found in carrion, marine epibenthos and desert stream communities after flooding (Lake et al. 1989).

### Taxa seasonality and canonical correspondence analysis

Temporary waters are seasonally colonised by aquatic insects (Fernando and Galbraith 1973). In the herein studied temporary pools, the spring and the autumn were the seasons that presented the highest richness and diversity. Prolonged flood events and climatic conditions favourable to the establishment of numerous species occurred in both seasons. A great number of immatures of many taxa were exclusively present during the spring and the autumn while other taxa were more abundant in these two seasons than in others. Temperature determines the seasonality of both species and habitat, since it directly affects the development and growth of the organisms, also indirectly affecting the quality and amount of available food, as well as the physico-chemical characteristics of the environment where the species will settle (Velasco et al. 1993).

As previously stated, pool hydroperiod plays a capital role in structuring the community. In fact, this variable was the first to appear in the CCA together with seasonality and DMTmi PW (a composed variable that synthesises the climatic conditions of the previous week). These variables explain most of the species distribution patterns throughout the year, also influencing the determination of the species composition. Among the variables incorporated to the statistical models, maximum depth seems to properly reflect the environmental availability, incorporating significance to annual and seasonal models. The degree of vegetation cover was also incorporated to some statistical models (annual and spring) reflecting its importance to aquatic insects. This variable is known to be related to numerous bionomic requirements of insects such as support for the displacement of the individuals (e.g. Archangelsky 1997), oviposition substrate (see Corbet 1980; Archangelsky 1997), and refuge against predation, or ambush place for predators (Schnack 1976; Corbet 1980), among other functions. Considering that the studied pools lack rooted or floating aquatic vegetation, the presence of lawn is an important source of vegetal substrate. The presence of filamentous algae also influences the composition of the community, not only as a food source for many of the species present (e.g. Oliva et al. 2002), but also because they provide refuge or oviposition sites to other species (e.g. Archangelsky 1997). Therefore, the incorporation of this variable into almost all of the statistical models is consistent. The few data obtained during the summer could not be ordered as a function of these environmental variables. The statistical analysis failed to relate any variable to the presence of species, mainly owing to the scarcity of records, which precluded the establishment of direct relationships between both data sets.

The taxa that functioned as indicator species in all statistical models (the larvae of chironomids and muscids, *Tropisternus* sp., *Rhantus* sp., and the larvae and adults of *Liodessus* sp. and *Sigara platensis*) can be considered as the characteristic species in these environments. The larvae of *Callibaetis* sp. and culicids, which were also frequent and abundant in these pools (except in the winter), must be included in the former group.

In agreement with published information, this study strongly suggests a clear link between habitat and community variability, with hydroperiod and seasonality the main factors in determining the community structure. Finally, considering that urban temporary pools (a) could reach values of richness similar to those of wild temporary habitats, (b) have the potential to act like corridors through the urban matrix to its dwellers, and (c) have an easy access for researchers, these aquatic environments are promising for the study of the ecological processes involving insect communities within cities.

## Figures and Tables

**Appendix:**
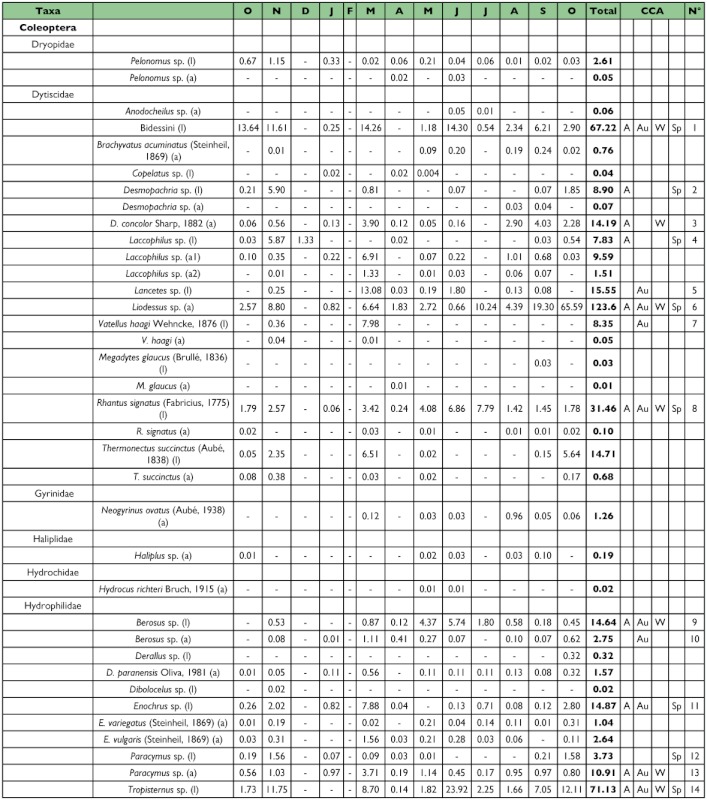
Monthly and total relative abundance (numbers of individuals per litre) of the insect taxa collected during the study period.

## Abbreviations

CCA: canonical correspondence analysis,
DMTmi PW: a variable that synthesises the climatic conditions of the previous week,
Hyd: hydroperiod is defined as the time elapsed since the pool formation until the corresponding sampling day,
THyd: total hydroperiod is the time elapsed from the formation of the pool until it dries completely
